# Pathophysiology behind prolonged whiplash associated disorders: study protocol for an experimental study

**DOI:** 10.1186/s12891-019-2433-3

**Published:** 2019-02-02

**Authors:** Anneli Peolsson, Anette Karlsson, Bijar Ghafouri, Tino Ebbers, Maria Engström, Margaretha Jönsson, Karin Wåhlén, Thobias Romu, Magnus Borga, Eythor Kristjansson, Hilla Sarig Bahat, Dmitry German, Peter Zsigmond, Gunnel Peterson

**Affiliations:** 1 Department of Medical and Health Sciences, Physiotherapy, Linköping University, Linköping, Sweden; 2 Center for Medical Image Science and Visualization (CMIV), Linköping University, Linköping, Sweden; 3 Department of Biomedical Engineering, Linköping University, Linköping, Sweden; 4 Pain and Rehabilitation Center, and Department of Medical and Health Sciences, Rehabilitation Medicine, Linköping University, Linköping, Sweden; 5 Department of Medical and Health Sciences, Division of Cardiovascular Medicine, Linköping University, Linköping, Sweden; 6 Department of Medical and Health Sciences, Radiological Sciences, Linköping University, Linköping, Sweden; 7 Herrgärdets Vårdcentral, Region Västmanland, Västerås, Sweden; 8 Landspitali University Hospital, University of Iceland, Reykjavik, Iceland; 9 Department of Physical Therapy, University of Haifa, Haifa, Israel; 10 Department of Neurosurgery and Clinical and Experimental Medicine, Linköping University, Linköping, Sweden; 11 Centre for Clinical Research Sörmland, Uppsala University, Eskilstuna, Sweden

**Keywords:** Whiplash injury, Neck, Spine, Chronic, Physiotherapy, Rehabilitation, Exercise therapy, Randomized, Follow-up study, Outcome, Physiopathology, Diagnostic imaging, MRI, Scan, Ultrasonography, Biomarkers

## Abstract

**Background:**

There is insufficient knowledge of pathophysiological parameters to understand the mechanism behind prolonged whiplash associated disorders (WAD), and it is not known whether or not changes can be restored by rehabilitation. The aims of the projects are to investigate imaging and molecular biomarkers, cervical kinaesthesia, postural sway and the association with pain, disability and other outcomes in individuals with longstanding WAD, before and after a neck-specific exercise intervention. Another aim is to compare individuals with WAD with healthy controls.

**Methods:**

Participants are a sub-group (*n* = 30) of individuals recruited from an ongoing randomized controlled study (RCT). Measurements in this experimental prospective study will be carried out at baseline (before intervention) and at a three month follow-up (end of physiotherapy intervention), and will include muscle structure and inflammation using magnetic resonance imaging (MRI), brain structure and function related to pain using functional MRI (fMRI), muscle function using ultrasonography, biomarkers using samples of blood and saliva, cervical kinaesthesia using the “butterfly test” and static balance test using an iPhone app. Association with other measures (self-reported and clinical measures) obtained in the RCT (e.g. background data, pain, disability, satisfaction with care, work ability, quality of life) may be investigated. Healthy volunteers matched for age and gender will be recruited as controls (*n* = 30).

**Discussion:**

The study results may contribute to the development of improved diagnostics and improved rehabilitation methods for WAD.

**Trial registration:**

Clinicaltrial.gov Protocol ID: NCT03664934, initial release 09/11/2018.

## Background

There is no consensus regarding the injury mechanism in complex prolonged whiplash associated disorder (WAD) cases. Often, tissue damage and physiological alterations are not detectable. To improve future rehabilitation, a greater understanding of the mechanisms underlying whiplash injury and their importance for treatment success is required. It is also important to investigate if pathophysiological changes can be restored by rehabilitation. Moreover, it is also important to realize that signs and symptoms of injury may abate, but adaptive dysfunctional patterns may develop that are secondary to the remaining functional deficits.

The cervical spine is heavily dependent on muscular support [1–6]. In particular, the deep neck muscles are important for cervical stability [1, 5, 7–9], but non-invasive diagnostic tools to measure impaired neck muscle function are lacking. Recently, ultrasound investigations have been used to simultaneously evaluate both deep and superficial neck muscle [5, 8, 9]. This non-invasive novel method can measure muscle deformation, which is the elongation or shortening of the muscle during real-time movement, and deformation rate, which is how fast the deformation occurs [10]. The results were very promising and revealed a model for impaired neck muscle function in WAD [8, 9]. Interactions between the muscle layers give complex data, but megavariate statistics and mathematical modelling of ultrasound images can be used in order to understand data [5, 8, 9]. Furthermore, there is scarce information about whether or not the impaired neck muscle function in chronic WAD can be restored by rehabilitation. Peterson et al. [5] published promising results from a smaller study regarding the different neck muscle layers, showing that three months of neck-specific exercises significantly improved ventral neck muscle interactions compared with staying on a waiting list. However, a larger study is needed to verify these results.

Fatty infiltration in the deepest dorsal neck muscle multifidus occurs in the early stage following a whiplash injury [11] and has also shown to be present in individuals with chronic WAD [12]. For the other neck muscles there is no or scarce information. A pilot study of five individuals with chronic WAD indicates that the fatty infiltration may be reversible after neck-specific exercises [13], but larger studies are needed. Moreover, the mechanism behind the development of fatty infiltration needs to be investigated. Yao & Gai [14] showed that Magnetic Resonance Imaging (MRI) T2 maps may be useful to study muscle inflammation and atrophy in the presence of muscle fat infiltration. Inflammation in neck muscles has, to our knowledge, never been investigated with MRI.

Changes in brain structure and function have been investigated with MRI/functional (f)MRI in chronic WAD in a few studies [15, 16]. However, the results need to be confirmed by other research teams and potential improvement after rehabilitation needs to be investigated. Also, the correlation with other clinical measurements needs to be investigated to learn about the clinical significance of such potential changes. Additionally, the WAD diagnosis and pain emanating from the neck must be confirmed with a clinical examination before inclusion according to the Quebec Classification criteria, which was not done in earlier MRI/fMRI studies.

Sterling et al. [17] indicated that inflammatory biomarkers in blood may play an important role in outcomes following acute WAD and may be related to the development of fatty infiltration in multifidus. No studies have investigated inflammatory biomarkers or stress biomarkers in chronic WAD or the association for outcomes after neck-specific exercises.

The muscle function and joint position of the cervical spine is important for postural control [18–20], and for interactions between the vision and vestibular systems [18, 20]. In other words, a good muscle function in the neck is necessary for good static balance and to avoid dizziness, pain and disability, as well as disturbed activity of daily life [18, 20]. In a few studies [21–23], the quantity and quality of joint position error and eye-neck coordination have been investigated in chronic WAD. Only Treleaven et al. [22] investigated the effect of neck-specific exercises (including a behavioural approach) on static balance and dizziness in chronic WAD and reported improvement in measures of dizziness, but not in balance, compared with general physical activity. The correlation between static balance/joint position error and self-assessed factors such as pain, disability and health needs to be further evaluated. The correlation between static balance/joint position error and pathophysiological factors such as neck muscle function, fatty infiltration and inflammation also needs to be investigated. In the present study, we use novel validated instruments with good precision that are easy to use in clinical practice.

In an ongoing randomized controlled multicentre trial (ClinicalTrials.gov Protocol ID: NCT03022812 and Peolsson et al. [24]), physiotherapist-led neck-specific exercise has previously [5, 22] shown to be effective for the current population and constitutes the control treatment for a new Internet-based neck-specific exercise treatment. For improved understanding not only of the best way of exercise distribution but also of pathophysiological mechanisms underlying whiplash injury and their importance for treatment success, investigation of such potential mechanisms is required. Improved knowledge of pathophysiological changes and their restoration potential may improve future diagnostics and rehabilitation.

## Aim

The projects aim to investigate muscle structure (neck and whole body muscle fatty infiltration, cross-sectional area, volume, inflammation), muscle function, brain structure and function related to pain, molecular biomarkers (for stress and inflammation), cervical kinaesthesia, postural sway and the association with pain, disability and other self-reported and physical outcomes in individuals with longstanding WAD before and after a neck-specific intervention [24]. Another aim is to compare individuals with WAD with healthy controls.

## Methods

### Design

**Fig. 1:**
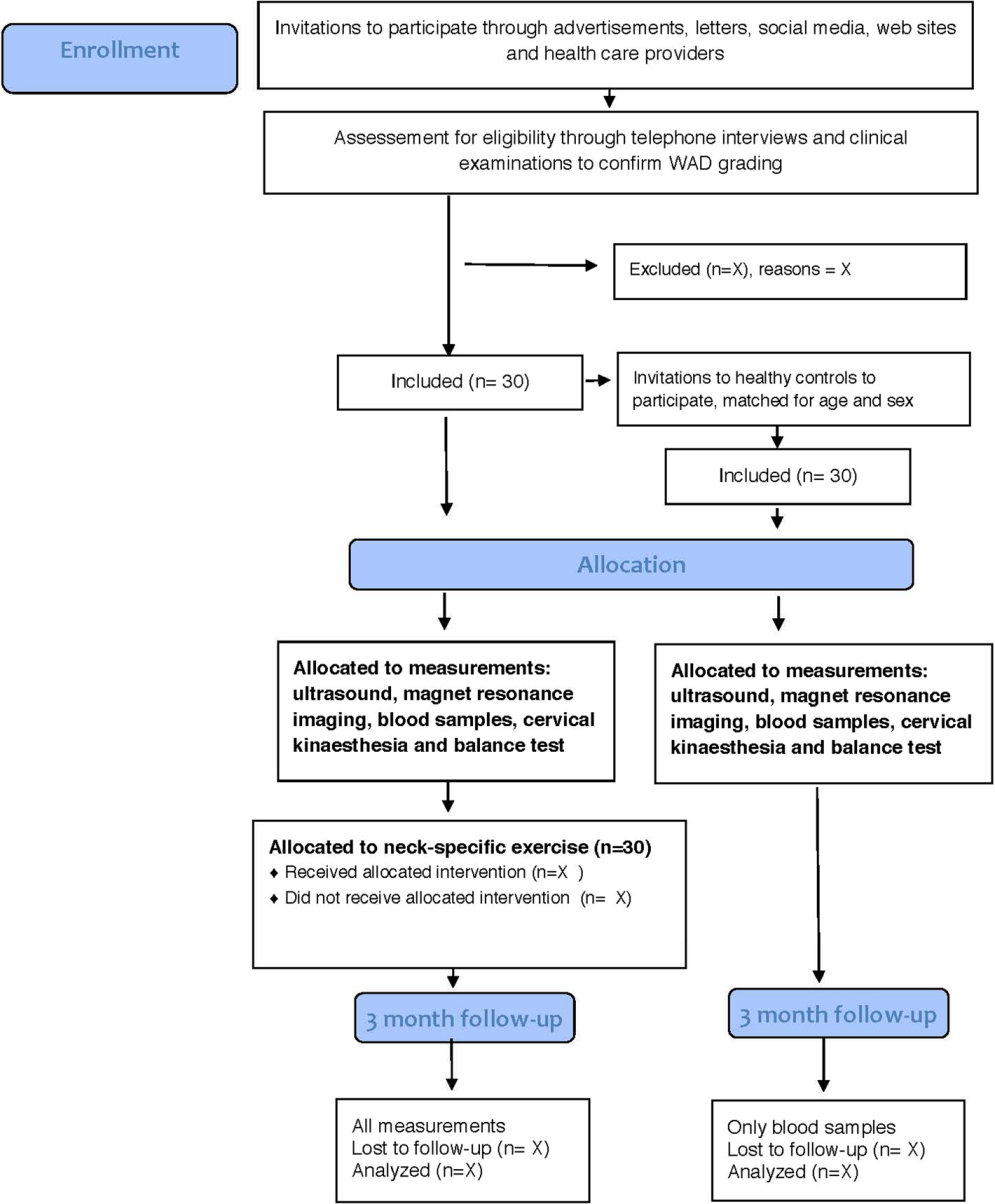
The Consort flow diagram

The projects, include experimental sub-group trials, each independent of the others, but using the same cohort to obtain data, in a prospective, multicentre, randomized controlled trial (RCT) with two parallel treatment arms conducted in outpatient care in Sweden according to a protocol established before recruitment started. (For further information, please see ClinicalTrials.gov Protocol ID: NCT03022812 and Peolsson et al. [24]) (Fig. 1). The study will consist of 140 patients in total (70 patients from each of the two groups) in the RCT, of which 30 will be asked to participate in the present prospective sub-group study consecutively. The sub-group trials include a 3 month follow-up and healthy individuals as matched control. If the RCT recruitment process is completed before all individuals in the sub-group study have been recruited the process will continue as described above. Independent physiotherapists in primary health care will distribute the treatment.

In the sub-group of individuals, measurements (MRI/fMRI, ultrasonography, blood and saliva sample, cervical kinaesthesia, static balance) will be performed before and after the end of interventions (three month follow-up). The physical measurements will be performed by independent specially trained test leaders, blinded to randomization.

Additionally, 30 neck healthy individuals without serious diseases matched for age and gender will be investigated consecutively.

The Regional Ethical Review Board in Linköping, Sweden (dnr 2016/135–31 and 2017/556–32) approved the projects. The protocol was registered before data collection started (Clinicaltrial.gov Protocol ID: NCT03664934).

### Study population

#### Inclusion criteria

The inclusion criteria for individuals with WAD are: chronic (> 6 months until 5 years since the accident) neck problems corresponding to WAD grades 2–3 [25] verified by clinical examination, shown to have exhibited a 20 mm minimum average estimated level of pain in the last week based on the visual analogue scale (VAS) [26] and a neck disability level higher than 20% on the Neck Disability Index (NDI) [27], of working age (18–63 years), within daily reach of a computer/tablet/smartphone and Internet, shown to have exhibited neck symptoms within the first week following the injury (i.e., neck pain, neck stiffness, or cervical radiculopathy), right-handed in addition to experiencing either equal-sided or dominant right-sided pain.

The inclusion criteria for healthy controls are: age and gender matched healthy individuals without neck pain or disability (VAS < 10 mm, NDI < 5%) who feel healthy overall, without known diseases.

#### Exclusion criteria

The criteria for exclusion for subjects of the study who suffer from WAD are as follows. Those who exhibit any of the following signs of head injury at the time of whiplash injury will be removed from consideration to take part in the study: amnesia before or after the injury, loss of consciousness, altered mental status (e.g., confusion, disorientation), focal neurological changes (changes in perceptions of smell and taste). Additional criteria for inclusion in the study are: previous fractures or dislocation of the cervical spine, a considerable degree of known or suspected physical pathology including myelopathy, spinal tumours, spinal infection, ongoing malignancy, cervical spine surgery, severe neck problems within their medical history which resulted in sick leave for more than a month in the year before the current whiplash injury, generalised or more overwhelming pain occurring elsewhere in the body presently, other illness/injury that may prevent full participation from being feasible, lack of ability to either understand or write Swedish, increased risk of bleeding, severe obesity (body mass index; BMI > 35), contraindications of MRI such as claustrophobia, metallic foreign bodies, pacemaker, cochlea implant, nerve stimulator and pregnancy.

Exclusion criteria for healthy controls are: earlier neck injury, recurrent neck pain, earlier treatment for neck pain, increased risk of bleeding, BMI > 35, contraindications of MRI [12].

### Recruitment and randomization

Information concerning the study will be provided by healthcare providers, reports in newspapers, social media, and the university’s website. Interested patients will contact the research team through the project website. Following the completion of a short survey on the website, a project team member (physiotherapist) will conduct a telephone interview and ask about the patient’s medical history. Arranging an appointment for a physical examination and an additional interview for the present sub-group study is set up as the last step to ensure that the criteria for study participation are met. If all the study criteria are met, and written and oral informed consent are confirmed, the patient will complete a written questionnaire and have physical measurements taken of neck-related functions [24]. Baseline measurements must be completed for inclusion [24].

Healthy individuals will be recruited consecutively among friends, family and staff who are not involved in the project at the university or the university hospital to match the age and gender of a patient.

### Intervention

Intervention for the patient group: The intervention consists of neck-specific exercises distributed in two different ways, twice a week at the physiotherapist clinic for three months (NSE group) or with four physiotherapy visits only combined with a web-based system (NSEIT group) [24]. Both groups will fill in an exercise diary. Participants in the present study will be recruited from both treatment arms consecutively, irrespective of randomization and handled as one neck-speck exercise group. Patients are asked to not seek other health care for their WAD (especially not physiotherapy) during the study period.

In the NSE group [24], patients will be offered an explanation and justification for the exercise which will contain rudimentary information concerning the aspects of the neck’s musculoskeletal anatomy that are relevant to the exercises given by the physiotherapist in order to motivate patient participation and aid in making them feel both safe and reassured. The patients undergo a 12-week training programme with a physiotherapist two days per week (a total of 24 times). Exercises are chosen from a clear and written framework of exercises. Included in the training programme are exercises for the muscles deep in the neck and continuation of endurance training of the neck and shoulder muscles. The exercises are adjusted one by one according to the physical conditions of the given individual and progressively increase in severity and dose. Pain provocation that is a result of exercise is not accepted, with the exception of cases entailing muscle soreness. The patient also has the option of performing the exercises at home. Upon the conclusion of the treatment period, the participants are encouraged to keep up the practice on their own time. In a previous RCT, the exercises were implemented with good results [22, 28–31].

In the NSEIT group [24], the information that participating patients will receive the same information and training programmes as the NSE group, but will entail four visits to the physiotherapist rather than 24. Exercises are introduced, guided to progress, and are followed up on to ensure correct performance. The exercises are performed and the vast majority of the information is provided with the help of Internet support that is completely external to the healthcare system. Photos and videos of the exercises (a clear stepwise progression) and information can be made available to interested parties by way of the web-based system. The system is programmed to automatically send a text message reminder in the event that the exercise diary is not completed in full. The time required for training is the same as in the NSE group, but without the patient having to go to the physiotherapy clinic. The Internet programme was developed by seasoned physiotherapists/researchers working alongside technicians and clinicians. Technicians are available to aid the participants should any technical difficulties arise over the course of the process.

### Variables and measurements

All measurements will be performed by experienced test-leaders, registered health-care personal and researchers that will monitor the data and guarantee high data quality. Adverse events and harms will be registered by the test-leaders. A research assistant will phone individuals that not appear. Data is part of the health secrets act (Swedish law) and will be stored at the Medical Faculty at the University and University Hospital. The project leaders will have access to the final trial dataset.

Patient background data include personal details, questionnaires and test results regarding pain, physical and psychological functioning, health and cost-effectiveness described elsewhere (ClinicalTrials.gov Protocol ID: NCT03022812, Peolsson et al. [24]). Additionally, questions about other diseases and medication will be asked. Measurements will be carried out at baseline and at a three month follow-up for the patient group when treatment ends.

For age- and gender-matched healthy volunteers, questions about background data such as age, gender, height, weight, physical activity, pain intensity, neck disability, other diseases and medication will be asked. Measurements will be carried out once, except for blood and saliva samples which will be collected twice (at baseline and repeated after three months).

#### MRI/fMRI

MRI will be used to investigate cross-sectional area, volume, fatty infiltration and inflammation of neck muscles, as well as whole body imaging. A Siemens Prisma 3.0 T will be used to acquire high-resolution neck images and whole body images. Water- and fat-separated images will be collected using three-dimensional, gradient-echo sequences. A 64-channel head coil and a coil blanket will be used in addition to the main body coil. The neck images will have an acquired resolution of 1.3*1.3*1.5 mm^3^ with a total scanning time of 5 min. The images will cover cervical segmental levels C2-TH1. A blinded investigator will perform semi-automatic segmentation of the ventral and dorsal neck muscles. The whole body images will have a resolution of 2*2*5 mm^3^ with a total scanning time of 6 min and neck to knee coverage. An automatic segmentation with quality assurance will be used to analyse the thigh muscles [32]. A T2 mapping sequence and a T2-weighted Dixon sequence, both sensitive to inflammation, will be collected for the ventral and dorsal neck muscles.

fMRI: Changes in the brain’s functional connectivity are assessed using a resting-state fMRI protocol with an echo planar imaging (EPI) sequence on a Siemens Prisma 3.0 T scanner with repetition time, TR, of 901 ms, iPAT (integrated parallel imaging techniques) = 2, and simultaneously multi-slice acquisition = 3, acquired voxel slice of 3 mm^3^, whole brain coverage, and a total of 10 min of acquisition time. Participants are instructed to lie still with their eyes closed during the fMRI scanning.

#### Ultrasonography

To investigate mechanical neck muscle activation, B-mode real-time ultrasonography movies will be obtained with a 12.0 MHz linear transducer in a longitudinal projection of the ventral (three layers) and dorsal (five layers) neck muscles at the fourth cervical segmental level during different neck and functional arm movements (during the entire exercise sequence [5, 8, 9] of repeated arm lift to 90° flexion, dynamic neck extension and rotation). The movies will be analysed afterwards with speckle-tracking software. An ultrasound of muscle results is an interference pattern of acoustic markers (speckle pattern) that can be analysed post-process via the use of an ultrasound movie sequence of images (AVI format). This process is formally known as speckle tracking analysis. During this process, a region of interest frame is set up above a standardised location within the speckle pattern of each muscle in the first frame of the video sequence [5, 8, 9]. The region of interest (ROI) tracks the unique speckle pattern one frame at a time over the course of the movie sequence. As the speckle pattern fluctuates in length between frames with muscle activity, the length of the ROI does as well. This change in the length of ROI represents muscle deformation [10]. Advanced multivariate mathematical modelling and data simulation will be used with the aim of developing a new diagnostic tool for impaired neck muscle function in WAD.

#### Biomarkers for inflammation and stress

The concentration of various biological markers for inflammation and stress in blood and saliva [33, 34] will be measured using multiplex immunoassay technology (Meso Scale Discovery, MSD). This multi-array technology enables the detection of up to 72 substances in multiplex format. Untargeted biomarker analysis will be performed using proteomics. This will be done by using mass spectrometry in combination with various separation methods such as two-dimensional gel electrophoresis (2-DE) and liquid chromatography (LC). A venous blood sample of about 10 ml is taken from the arm. The saliva sample is taken 15 min after washing the mouth with water by placing a cotton swab (Salivette) in the mouth for 3 min. All samples will be unidentified (marked with a code number). 2-DE instruments in combination with digitizing camera and special software (PDQuest, Bio Rad) for protein separation and quantification are available at the PAINOMICS® laboratory (Linköping University). The laboratory is also equipped with a MESO QUICKPLEX SQ 120 instrument (Meso Scale Discovery, Maryland, USA). EASY-nLC II (Thermo Scientific) combined with LTQ Orbitrap Velos Pro hybrid mass spectrometer (Thermo Scientific) with a nano-electrospray source available at the Core Facility at the Medical Faculty, Linköping University will be used.

#### Cervical kinaesthesia

A neck gear/plastic helmet with a 3D accuracy orientation sensor that tracks the cervical position sense in space, “the butterfly test” (by Eythor Kristjansson and co-workers, Reykjavik, Iceland) [21, 23] will be used. Participants will sit in a good postural position (slight support for the low back), thoracic spine fixated at Th4 level, neck in neutral position, hands on thighs, thighs apart, 90 cm in front of a computer screen, with the helmet on their head. Through neck movements, the patient will control a marker and try to follow the “butterfly” moving in specific patterns on the screen [21, 23]. The system measures three subsets of neck proprioception in real time, while the participants are moving their head and neck: amplitude accuracy in millimetres; directional accuracy in percent (%) of the total time used to perform the trial (time on target, undershoots versus overshoots); smoothness (ease versus jerkiness) of movements via unitless index from 0 to 5. Three incrementally difficult movement patterns appear on the screen, which the participants are required to follow with movements of their head and neck. Each pattern is repeated three times in random order, and a total of nine movement patterns are performed. Before each pattern the system counts down from 3 to 1. Participants will be told to move their head and neck once in a left to right rotation between patterns and then go back to their neutral neck position before the next pattern appears on the screen. Participants are asked do their best, but will not otherwise be encouraged. A pre-test trial will be performed to familiarize participants with the test procedures.

The whole cervical spine active range of motion is also measured in the software. Both quantity and quality (through associated movements) of cervical movements are registered.

#### Static balance

In the present study, static balance will be assessed using an iPhone 8 customized application for postural sway assessment developed at the University of Haifa, Israel, for research [35]. The iPhone will be attached to the pelvis on a waist belt so that the iPhone sensors are positioned at S2 level, representing the centre of gravity [35].

Static balance will be assessed in two positions: single leg open eyes and double stance closed eyes, each to be repeated three times.

During the single leg stance there will be a free choice of preferred standing leg. The raised leg will not allowed to touch the standing leg, and will be in a slightly flexed hip and knee position. Arms will be crossed over the chest with eyes open. In double stance testing, hands will be on hips with eyes closed. Participants will start with a 10 s warm-up session for both positions before measurements to ensure that they understand the instructions [35]. There will be a 30 s rest in between repetitions, and a 60 s rest in between positions. Participants will stand barefoot as long as possible, up to a maximum of 40 s. Each session will start with the app counting down from 5 to 1, followed by a beep to signal the start, and beep to signal the end. The tester will not encourage the participants during the tests.

Postural sway data will be analysed by a blinded data analyst, remote from the set-up location. Postural sway analysis will provide measures of pathway and 95% ellipsoid area in x and y planes [35].

### Ethical considerations

The projects were approved by the Regional Ethical Review Board in Linköping, Sweden before the projects started. Neck-specific exercises are carried out according to the best scientific evidence, and have demonstrated good efficacy in long-standing WAD in a previous study [22, 28–31]. The exercises are adjusted individually from a framework of exercises. Participants are not expected to experience danger or harm. All entailed physical tests are concretely established and are already used in clinical practice, although the way of analysing the data will add to current knowledge. All physical tests will be performed by a registered health care professional with the necessary knowledge and authorization to perform the tests. Participants with WAD are included after interviews and thorough clinical examinations by registered health care personnel. Controls regard themselves to be healthy and are included after thorough interviews. All participants will give their signed, informed consent. Additional clinical MRI imaging of the neck is obtained for individuals with WAD and analysed by a radiologist, and the results are communicated to the patient by a neurosurgeon. The results will be presented at group level, and no connection to the individual person can be made. All data are subject to the Official Health Secrecy Act.

### Data analysis and statistics

For the RCT, these are described elsewhere (ClinicalTrials.gov Protocol ID: NCT03022812 [24]).

Power calculation cannot be carried out for the pathophysiology data as the effect sizes are unknown after rehabilitation. Our earlier studies have shown that 30 individuals are enough for comparisons between individuals with health problems and healthy controls [5, 6, 8, 9, 12, 21–23, 33–35].

Data will be analysed using parametric or non-parametric statistics depending on the type of data. The best specific statistical tests to use depends on the type of data, normal distribution and whether the analysis is between groups or over time (the patient group).

Ultrasound speckle tracking analyses (deformation and deformation rate) and inflammatory and stress biomarkers will potentially be analysed with multivariate methods such as principal component analysis (PCA) and orthogonal partial least squares (OPLS). Additionally, ultrasound data will potentially be analysed with mathematical mechanistic modelling that allows for a description of the different functions or mechanisms underlying the behaviour of the muscles. Each mechanism is described by a mathematical equation and simulated over time. This allows us to compare the output of the model to the measured data and test whether a specific hypothesis can explain the behaviour of the data, as well as making predictions that can be used for diagnosis or planning experimental setup.

Resting state fMRI data will be analysed using the CONN functional connectivity toolbox (http://www.nitrc.org/projects/conn). Here, pre-post intervention changes in functional connectivity will be investigated with ROI-to-ROI (region of interest) and seed-to-voxel analysis focusing on brain networks involved in the perception of pain, e.g. the salience network with main hubs in the insula and the anterior cingulate cortex. We will also investigate functional connectivity in relation to the frontal eye fields, an area of the brain that is involved in voluntary and saccadic eye movements.

### Timetable

The project started on October 4, 2018. The inclusion period is expected to be finalized around December 2019. Thereafter, participants with WAD will be followed for another three months. Blood and saliva samples will be collected twice (at baseline and repeated after three months) for healthy individuals as well.

## Discussion

The clinical advantage of the projects is great because individuals with long-standing grade 2 and 3 WAD experience disability and suffering. The present study is novel, unique and multi-professional, and is of great significance for people who suffer following a whiplash injury, as well as for society. The mechanisms behind chronic WAD and whether pathophysiological findings can be restored are largely unknown. Understanding the mechanisms and improved diagnostics are important key factors for future improved rehabilitation. The long-term goals of the projects are to optimize treatment plans for patients, which will improve their health and social participation. Some results may also be generalized to other neck pain conditions.

## Trial limitations

These are rather new areas under technical innovation and development implemented in individuals with longstanding WAD, which makes the power analyses hypothetical. Previous data from reliable larger studies from which to count the sample size are not avaliable, especially not after a neck-specific exercise period for those with longstanding pain after a whiplash injury. The projects are novel and of great importance, and may therefore be seen as pilot studies. However, our earlier studies have shown that 30 individuals are enough for comparisons between individuals with health problems and healthy controls.

## Abbreviations

2-DE: two-dimensional gel electrophoresis
3D: three dimensional
AVI: Audio Video Interleaved
BMI: body mass index
B-Mode: brightness mode = a two-dimensional ultrasound image display composed of bright dots representing the ultrasound echoes
CONN: CONN is an open-source Matlab/SPM-based cross-platform software for the computation, display, and analysis of functional connectivity Magnetic Resonance Imaging
EPI: echo planar imaging
fMRI: functional magnetic resonance imaging
iPAT: integrated parallel imaging
LC: liquid chromatography
MHz: Mega hertz
MRI: magnetic resonance imaging
NDI: neck disability index
NSE: neck-specific exercise
NSEIT: neck-specific exercise with internet support
OPLS: orthogonal partial least squares
PCA: principal component analysis
RCT: randomized controlled study
ROI: region of interest
S2: second sacral vertebra
T: tesla
Th: thoracic spine
TR: repetition time
VAS: visual analogue scale
WAD grade 2: whiplash associated disorders with local (neck) musculoskeletal signs according to the Quebec task force (QTF) classification system
WAD grade 3: whiplash associated disorders with local (neck) and neurological signs according to the Quebec task force (QTF) classification system
WAD: whiplash associated disorders
